# Burning Mouth Syndrome as a Central Pain Disorder: A Case Study Demonstrating Response to Occipital Nerve Block Treatment

**DOI:** 10.3390/dj14020081

**Published:** 2026-02-02

**Authors:** Shachar Zion Shemesh, Paz Kelmer, Lior Ungar

**Affiliations:** 1 Department of Neurosurgery, Sheba Medical Center, Ramat Gan 52621, Israel; pazkelmer92@gmail.com (P.K.); lior.ungar@sheba.health.gov.il (L.U.); 2 Gray School of Medicine, Tel Aviv University, Tel Aviv 6139001, Israel; 3 Dina Recanati School of Medicine, Reichman University, Herzelia 4610101, Israel

**Keywords:** Burning Mouth Syndrome, occipital nerve block, trigeminocervical convergence, central sensitization, subacute neuropathic pain

## Abstract

**Background:** Burning Mouth Syndrome (BMS) is a chronic orofacial pain condition characterized by a burning sensation in the oral cavity without identifiable lesions. It predominantly affects women (especially postmenopausal) but can occur in men. BMS is considered a multifactorial neuropathic pain disorder involving both peripheral small-fiber neuropathy and central dysregulation, often accompanied by taste alterations (dysgusia) and xerostomia despite normal oral exams. Treatment is challenging, with modest responses to agents like clonazepam, tricyclic antidepressants, or gabapentinoids. **Observations:** We present a 67-year-old male with recalcitrant primary BMS who showed complete remission temporally associated with occipital nerve blockade, likely affecting central trigeminocervical pathways. Initial therapy with amitriptyline (25 mg) and gabapentin (900 mg/day) yielded ~30% pain relief. Given suspected central sensitization, greater and lesser occipital nerve (GON) blocks were administered in series. After the first, second, and third ON blocks, pain was reduced by ~50%, 80%, and 100%, respectively. Remission persisted at one-year follow-up under continued medications. A mild recurrence (~20% of baseline pain) responded fully to a fourth GON block, maintaining another year of pain-free status. **Lessons:** This case underscores the complex central mechanisms in BMS and illustrates that modulating central pain circuits via occipital nerve blockade, through trigeminocervical convergence mechanisms, without direct trigeminal intervention. We discuss the diagnostic challenges of BMS, the rationale of occipital neuromodulation, and how this novel therapeutic strategy compares with current literature, supporting the hypothesis of central sensitization in BMS.

## 1. Introduction

Burning Mouth Syndrome (BMS) is a chronic idiopathic pain disorder defined by unremitting burning sensations in the oral mucosa, typically involving the tongue and/or lips, in the absence of any visible lesions or systemic causes [1]. It is a diagnosis of exclusion that often requires ruling out secondary causes such as nutritional deficiencies, candidiasis, xerostomia, or neuropathies. The International Association for the Study of Pain classifies primary BMS as a distinct entity characterized by daily burning pain for ≥3 months without clinical abnormalities. BMS is relatively uncommon in the general population (estimated prevalence ~ 0.7%) but disproportionately affects middle-aged and older adults, with up to 10–15% of postmenopausal women experiencing BMS [2]. Patients often report concomitant taste changes (metallic or bitter dysgeusia) and a sensation of dry mouth, despite normal salivary gland function [3]. Psychological distress and sleep disturbances are commonly associated [4].

The pathophysiology of BMS is complex and not fully understood. Emerging evidence points to BMS as a neuropathic pain condition with both peripheral and central components [5]. On the peripheral side, numerous studies have demonstrated small fiber neuropathy involving the trigeminal system. For example, intraepithelial nerve fiber density in the tongue can be reduced by 30–60% in BMS, and pain-related receptors such as TRPV1 and P2X3 are upregulated in affected oral tissues [6]. These findings indicate a peripheral nociceptive dysfunction. At the same time, central nervous system changes are evident: neurophysiological studies show deficient pain-inhibitory mechanisms and altered brainstem reflexes in BMS [7]. Functional imaging has also suggested abnormal central processing of pain and taste. Taken together, BMS is recognized as a multifactorial neuropathic disease with “dual involvement” of peripheral small fibers and central sensitization [5]. In fact, experts propose that patients with BMS may be subdivided into a predominantly peripheral subtype versus a central subtype- the former might respond to local treatments, whereas the latter is thought to involve centralized pain and often correlates with psychological comorbidities [8,9].

The management of BMS is challenging due to its unclear etiology and variable response to therapies [3]. No single treatment provides consistent relief in all patients, and many interventions are used empirically. Topical clonazepam and capsaicin have demonstrated benefit in randomized trials [10]. Systemic clonazepam, alpha-lipoic acid, and cognitive-behavioral therapy have also demonstrated benefits in some studies [11,12]. Nonetheless, many patients obtain only modest or incomplete relief [12]. This therapeutic refractory nature suggests that novel approaches targeting the underlying mechanisms are needed.

There is growing interest in neuromodulatory interventions for refractory orofacial pain conditions. In BMS, the observation that some patients experience temporary pain relief from local anesthetic blocks of the lingual or mandibular nerve has provided insight into peripheral vs. central mechanisms [8]. Beyond local trigeminal blocks, other regional neuromodulation techniques have been explored. Walega et al. reported a case of recalcitrant BMS successfully treated with bilateral stellate ganglion block (cervical sympathetic blockade) [13], hypothesizing a role for sympathetic nervous system involvement in maintaining oral pain. This suggests that interruption of certain neural pathways outside the trigeminal nerve proper can modulate BMS pain [14].

One such pathway is the trigeminocervical system. It is well known from headache literature that sensory afferents from the upper cervical spine (C1–C3) converge with trigeminal nerve fibers in the trigeminal nucleus caudalis of the brainstem, forming the so-called trigeminocervical complex. In particular, the ophthalmic division (V1) of the trigeminal nerve and the upper cervical nerves (including the greater occipital nerve from C2) share common second-order neurons in the caudal trigeminal nucleus. This anatomical convergence means that cervical nociceptive input can influence trigeminal pain pathways (and vice versa). Clinically, this is evidenced by referred pain patterns and the efficacy of cervical interventions on facial pain. Greater occipital nerve (GON) blocks, for example, are routinely used to treat migraine and cluster headaches and have also shown benefit in certain craniofacial neuralgias. The pain relief from GON block in trigeminally innervated regions is thought to result from inhibition of afferent traffic into the trigeminal nucleus caudalis, thereby dampening central sensitization within the trigeminocervical complex. In essence, by anesthetizing or modulating the C2 fibers (occipital nerve), one can induce a central neuromodulatory effect that quiets hyperactive trigeminal nociceptive circuits.

## 2. Case Description

**Patient Profile:** A 67-year-old right-handed man with a significant medical history of ischemic heart disease (status post seven coronary stents), type 2 diabetes mellitus, hypertension, dyslipidemia, benign prostatic hypertrophy, and gastroesophageal reflux disease developed a spontaneous burning pain in his mouth. The oral pain had an insidious onset and progressively worsened over several months, becoming constant in nature. He described a bilateral burning sensation primarily on the anterior tongue and hard palate, accompanied by a persistent bitter metallic taste (dysgeusia). The symptoms were present daily, often peaking in the late afternoon and evening. He also noted subjective dry mouth, though he was able to produce normal saliva. No trigger factors (such as food intake, hot/spicy foods, or dental products) consistently altered the pain, but he observed that stress and anxiety exacerbated the burning sensation.

The patient had no history of neck pain, occipital neuralgia, cervicogenic headache, or other craniocervical pain syndromes. There was no tenderness over the greater or lesser occipital nerves on examination. His pain was strictly localized to the oral cavity (anterior tongue and hard palate) without any occipital, neck, or facial radiation.

**Diagnostic Work-up:** The patient underwent a thorough evaluation to exclude secondary causes of oral burning. Oral examination by both a dentist and an oral medicine specialist found no lesions or abnormalities of the tongue, mucosa, or dentition. There was no glossitis, candidal infection, ulceration, or galvanic dental issue. Salivary flow was within normal limits. Laboratory tests, including complete blood count, fasting glucose (which was controlled on his medications), HbA1c, vitamin B12, folate, iron studies, thyroid function, and autoimmune screening, were unremarkable. An otolaryngological exam of the oral cavity and oropharynx was normal. Given his complaint of taste disturbance, an MRI of the brain with gadolinium contrast was obtained to rule out any central lesion (such as a brainstem or cranial nerve tumor). The MRI, with dedicated high-resolution sequences through the cerebellopontine angle and internal auditory canals, showed no abnormality of cranial nerves VII (facial nerve, which carries chorda tympani taste fibers) or VIII, and no intracranial lesions. This comprehensive negative work-up supported the diagnosis of primary (idiopathic) Burning Mouth Syndrome [1].

**Initial Management:** The patient’s BMS was managed conservatively at first. He was started on low-dose amitriptyline 25 mg at night for neuromodulation and analgesia, with caution given his cardiac history (the dose was kept low to mitigate any anticholinergic or arrhythmic risk). In addition, gabapentin was titrated to 300 mg three times daily (900 mg/day) over several weeks. This combination of a tricyclic antidepressant and an anticonvulsant is often used empirically for neuropathic pain, including atypical facial pain, and was intended to reduce oral burning discomfort. After two months on this regimen, the patient reported approximately 30% improvement in pain intensity. The burning quality persisted, but he had more tolerable moments and slight relief in the dysgeusia. However, the pain remained moderate and disruptive to his quality of life, and he continued to require clonazepam 0.5 mg as needed for anxiety related to the symptoms (though clonazepam was not used in a high enough dose specifically for BMS pain control). Given the suboptimal response and the known challenges in treating BMS [3], we explored additional interventions.

**Neuromodulatory Intervention:** Based on the hypothesis of a centralized pain mechanism, a decision was made to attempt a trigeminal-occipital nerve block approach. After discussing risks, benefits, and the experimental nature of this strategy with the patient, we proceeded with a greater and lesser occipital nerve block series. Each injection was performed using real-time ultrasound guidance (high-frequency linear transducer) to ensure accurate nerve localization and minimize complications. Both the greater and lesser occipital nerves were targeted bilaterally at the level of the superior nuchal line. Both the greater and lesser occipital nerves were targeted bilaterally. Each nerve received 3 mg of betamethasone (Celestone) diluted in 2 mL of 1% lidocaine, for a total of four injections per session. The patient tolerated the procedure well, with only minor local soreness. Within one week, he reported approximately 50% reduction in his tongue burning (patient self-assessment) and was able to eat spicier foods with less discomfort. This improvement was sustained for one month until the second block. Encouraged by this partial relief, we repeated the ON blocks one month later. After the second injection (performed in the same manner bilaterally), his pain further decreased, reaching about 80% improvement in intensity and frequency (patient self-assessment). This effect was maintained for approximately one month. The dysgeusia also diminished correspondingly. A third ON block was administered another month thereafter, resulting in a complete remission of the oral burning pain. Following the third injection, the patient described his mouth as feeling “normal” for the first time in over a year- he had no significant burning even late in the day, and food taste was nearly back to baseline. He continued on the amitriptyline and gabapentin during this period, with plans to taper these medications if pain relief was maintained.

**Follow-Up:** At a routine follow-up 12 months after the third block, the patient remained essentially pain-free. He had resumed a full diet without restrictions and reported only occasional mild oral dryness. His mood and sleep had improved markedly now that the chronic pain was in abeyance. We opted to maintain him on a low dose of amitriptyline (10–25 mg) for another year and gradually wean gabapentin, given his stable remission. Around 12 months after achieving remission, the patient noted a slight return of a burning sensation (approximately 20% of the original pain level by his estimation). This mild relapse prompted a fourth ON block, which again abolished the symptoms completely. As of his last follow-up (24 months from initial presentation), he continues to be pain-free without any oral burning or taste disturbance. He remains on a minimal dose of amitriptyline (10 mg nightly) and uses no daily analgesics. No adverse effects from the occipital nerve blocks were observed aside from transient injection site tenderness. Table 1 summarizes the timeline of interventions and symptom progression in this case. To the best of our knowledge, occipital nerve blockade has not previously been reported as a therapeutic intervention for Burning Mouth Syndrome. While occipital nerve blocks are well established in the management of primary headache disorders and certain craniofacial pain syndromes, their application in BMS has not been systematically described in the literature.

## 3. Discussion

This case illustrates a unique treatment approach for Burning Mouth Syndrome and sheds light on the potential central mechanisms underlying this enigmatic pain disorder. The patient’s dramatic improvement with occipital nerve blocks suggests that his BMS was maintained by central nociceptive circuits amenable to modulation, rather than purely peripheral oral pathology [15]. In effect, by “turning down” input at the level of the C2 occipital nerves, we were able to alleviate pain in the trigeminal distribution (the oral cavity). This outcome provides clinical evidence supporting the concept of trigeminocervical convergence playing a role in idiopathic facial pain.

BMS has long been challenging to diagnose and manage due to its multifactorial nature. In our patient, extensive evaluations confirmed that we were dealing with primary (essential) BMS, consistent with the typical presentation of bilateral burning oral pain and taste changes without any lesion [16,17]. The epidemiology of our case—a male in his late 60 s—is slightly atypical given the female predominance of BMS [2], but not unheard of. It is noteworthy that despite significant comorbidities (diabetes and vascular disease), no secondary cause for his oral burning (such as neuropathy from diabetes) was evident, which aligns with primary BMS being a diagnosis of exclusion. His symptoms being modulated by emotional stress fits with observations that BMS pain can be exacerbated by anxiety or depression, and many patients with BMS have underlying affective disorders [4]. This mind-pain interaction further hints at a central augmentation of pain perception.

Several clinical features support classification as centrally mediated BMS: (1) bilateral distribution, (2) absence of local triggers, (3) constant burning pattern, (4) associated dysgeusia, (5) partial response to systemic neuromodulators (~30%), (6) stress-related exacerbations, and (7) normal peripheral examination. While consistent with centralized pain, definitive subtyping would require specialized testing (quantitative sensory testing, diagnostic nerve blocks), which were not performed.

The partial response to amitriptyline and gabapentin in this case is in line with the literature, which suggests these systemic agents can provide modest relief in some patients with BMS, but often not complete resolution [9,12]. Tricyclics and gabapentinoids target neuropathic pain by enhancing descending inhibition or dampening neural hyperexcitability, so the fact that our patient improved by ~30% indicates a neuropathic component was present, yet the persistence of significant pain implied that stronger or different neuromodulation was necessary. Interestingly, one of the more effective evidence-based treatments for BMS is topical clonazepam (anxiolytic and central suppressant) applied to the tongue/oral mucosa, which leverages both local and central mechanisms. Our patient did not use topical clonazepam, but his need for occasional systemic clonazepam for anxiety could have provided minor additional relief. In general, the variable efficacy of pharmacotherapies underscores the heterogeneity of BMS mechanisms.

An important limitation of this case is the concurrent use of systemic medications throughout treatment and follow-up. The patient remained on amitriptyline 25 mg and gabapentin 900 mg/day during the nerve block series and subsequent remission period. While these medications alone provided only approximately 30% relief during the initial two-month trial, it is possible that their continued use contributed to the eventual complete remission through cumulative neuromodulatory effects, synergistic interaction with nerve block-induced changes, or delayed therapeutic response. The temporal correlation—with dramatic improvement occurring specifically after each nerve block rather than during the medication-only period—suggests the blocks played a significant role. However, we cannot definitively exclude medication contribution or synergy. Future studies should include medication washout periods or control groups to isolate the effect of nerve blocks, though ethical considerations may limit such designs in patients with refractory pain.

The combination of local anesthetic (lidocaine) with corticosteroid (betamethasone) in our nerve blocks warrants discussion. This combination is standard practice in pain management and was chosen for several potential mechanisms: the local anesthetic provides immediate neural blockade and may disrupt maladaptive pain circuits, while the corticosteroid may provide prolonged anti-inflammatory effects on the nerve and surrounding tissues, extended duration of neural membrane stabilization, and reduction in neurogenic inflammation. The sustained benefit observed (well beyond the duration of local anesthetic action) suggests a prolonged effect, possibly from the steroid component or from lasting neuromodulatory changes (synaptic plasticity) induced by the temporary blockade. We cannot determine from this case whether a local anesthetic alone would have achieved similar results. Some headache literature suggests anesthetic-only GON blocks can provide prolonged relief, while other studies favor the addition of corticosteroids. Future comparative studies using anesthetic-only versus combination blocks would help clarify the relative contributions of each component.

The success of the greater occipital nerve blocks in our patient highlights the role of the central nervous system in sustaining BMS pain. The GON supplies sensation to the posterior scalp (occiput), not the mouth, so any effect on oral pain must occur through central connections. The trigeminocervical complex provides a plausible pathway: second-order neurons in the spinal trigeminal nucleus receive convergent input from both trigeminal afferents (face, oral cavity) and upper cervical afferents (including GON). In conditions like migraine or cluster headache, blocking the ON can reduce pain partly by inhibiting excitatory input to these shared pathways [18]. We postulate that a similar phenomenon occurred in BMS. By anesthetizing the ON (and possibly reducing inflammation via the steroid), we likely diminished the overall afferent traffic and hyperactivity in the trigeminal nucleus caudalis, thereby “quieting” the central sensitization that was driving the oral burning sensation [19]. This is consistent with known mechanisms: GON block has been reported to produce relief even in trigeminal neuralgia and other facial neuralgias, despite the GON being anatomically distinct, due to this convergence at the brainstem level. Our case extends this concept to BMS, which has not been widely reported before.

While the trigeminocervical convergence mechanism is well-established in headache literature, evidence in non-headache orofacial pain remains limited. Most GON block data come from headache trials. Extrapolation from headache to BMS is theoretically sound based on shared neuroanatomy, but remains largely untested. Our case provides preliminary clinical data, but the mechanism remains hypothetical for BMS. The application of occipital nerve blocks to BMS remains experimental. It supports the idea that primary BMS (especially the centrally mediated subtype) might be considered a craniofacial analog of centralized pain syndromes, amenable to treatment approaches beyond the immediate site of pain.

Comparing our approach to the existing literature: previous reports have explored peripheral nerve interventions for BMS with mixed results. Grémeau-Richard et al. (2010) [6] found that a lingual nerve block with lidocaine could transiently abolish pain in a subset of patients with BMS (approximately half), but the pain promptly recurred when the anesthetic wore off [8]. This suggests that while peripheral input (from lingual nerve small fibers) contributes to pain, sustained relief was elusive without addressing central sensitization. Topical anesthetics (like benzocaine gels) similarly often fail to provide lasting benefit in true BMS. In another notable case report, Walega et al. (2014) [5] achieved significant relief in a BMS patient using bilateral stellate ganglion blocks [13,20]. The stellate ganglion block, which anesthetizes the sympathetic outflow to the head/neck, is thought to help neuropathic pain by reducing sympathetically maintained pain and improving regional blood flow. The positive outcome in that case hinted at a possible role of dysregulated sympathetic activity or central pain facilitation in BMS. Our use of occipital nerve block works through a different route (somatic afferent modulation), but both approaches converge on the final common pathway of reducing central pain signaling. It is noteworthy that in both the stellate ganglion case and ours, the pain relief was long-lasting after a series of injections, well beyond the acute duration of local anesthetic action. This implies a reset of neural circuitry—perhaps breaking a “pain cycle” or inducing synaptic plasticity that restores a more normal sensory processing state.

Our patient’s course also raises interesting questions about maintenance therapy and recurrence. After the third ON block, he enjoyed a full year of remission on conservative doses of medication. The slight relapse at one year may indicate that whatever central sensitization had been curtailed was slowly building up again (possibly triggered by new stressors or metabolic changes). The successful repeat block then re-established the pain-free state. This pattern is reminiscent of how certain headache disorders are managed: for example, patients with chronic cluster headache may receive periodic occipital nerve blocks to stay in remission. It suggests that for some patients with BMS, occasional interventional treatments could be a viable strategy to maintain long-term control, in combination with pharmacotherapy. In the future, if such an approach is validated, patients who respond to blocks might even consider neuromodulation implants (e.g., occipital nerve stimulators) for continuous pain control, analogous to their use in refractory headache syndromes. Such speculation needs clinical study, but it is an intriguing direction opened by this case.

Importantly, the success of the occipital nerve blockade in this case may relate to the subacute nature of the condition. It is possible that central sensitization had not yet become fully established and that no irreversible small fiber degeneration was present. In more chronic cases, particularly those with long-standing neuropathic changes, similar interventions might be less effective.

From a safety and tolerability standpoint, greater occipital nerve blocks are relatively low-risk, especially when performed with careful technique. Aside from minor local pain or hematoma, serious complications are rare. Our patient had no complications over four procedures. This safety profile is important given that many patients with BMS are older and may have comorbidities (as in our case), making invasive neurosurgical procedures undesirable. An occipital block is a simple outpatient intervention that, if effective, offers a favorable risk-benefit ratio compared to systemic high-dose medications or experimental surgeries.

Another limitation is the absence of validated outcome measures. Pain improvement percentages were based on patient self-report without standardized instruments such as the Visual Analog Scale (VAS), Brief Pain Inventory (BPI), or quality of life measures. This limits comparability with other studies and introduces potential recall bias.

It must be emphasized that this is a single case report, and one cannot generalize that all patients with BMS will respond similarly to occipital nerve blockade. BMS is a heterogeneous condition—some cases may be predominantly peripheral (e.g., related to undetected small fiber neuropathy of the lingual nerve or glossopharyngeal nerve) and others more central. Identifying which patients have a central sensitization phenotype could help target therapies like occipital blocks. Clinical clues might include the degree of pain independence from local stimuli, presence of other central sensitization symptoms, or lack of response to localized treatments. In our patient, the bilateral, non-triggered nature of pain and partial response only to systemic drugs suggested a central mechanism, which guided us toward a central neuromodulation approach. In practice, a diagnostic trial such as a one-time lingual nerve anesthetic block could be used: if it fails to significantly relieve pain, that might predict a central-maintained BMS, which could be a candidate for treatments as we employed.

## 4. Conclusions

This case report provides key insights into BMS management:

1. Novel therapeutic approach: This represents the first reported case of BMS successfully treated with occipital nerve blockade, extending trigeminocervical neuromodulation beyond headache disorders to refractory orofacial pain.
2. Central pain mechanism: Successful trigeminal pain modulation without direct trigeminal intervention supports the hypothesis that BMS is maintained by central sensitization within the trigeminocervical complex.
3. Clinical efficacy: The serial occipital nerve blocks produced progressive relief (50% → 80% → 100%) following partial pharmacotherapy response, with sustained remission at 24-month follow-up.
4. Timing considerations: The favorable response in this subacute case suggests that early neuromodulatory intervention may prevent irreversible central sensitization or small fiber degeneration.
5. Safety profile: Occipital nerve blockade offers a low-risk outpatient option, particularly valuable for elderly patients with comorbidities.
6. Future directions: While limited by a single-case design, this proof-of-concept warrants prospective trials to validate the approach and identify response predictors in larger cohorts.

Ultimately, personalized mechanism-based management tailored to peripheral versus central contributions will improve outcomes in this challenging condition.

## Figures and Tables

**Table 1:** Treatment Timeline and Symptom Response in a BMS Patient.

Timepoint	Intervention	Outcome on BMS Symptoms
Baseline (Diagnosis)	BMS was diagnosed after negative work-up.	Severe bilateral burning mouth pain; dysgeusia; no structural lesion.
Month 0–2	Amitriptyline 25 mg qHS + Gabapentin 900 mg/day.	~30% reduction in pain intensity; partial relief of burning, but moderate pain persists.
Month 3	1st Occipital Nerve block (bilateral, bupivacaine + steroid).	~50% pain relief; burning sensation significantly less frequent and intense.
Month 4	2nd ON block (repeat bilateral injection).	~80% pain relief; only mild residual burning; improved taste sensation.
Month 5	3rd ON block (repeat bilateral injection).	100% pain relief (complete remission of burning pain and dysgeusia).
Month 6–12	Maintenance on medications; no new interventions.	Pain-free, normal oral sensation maintained.
12 Month Follow-up	Mild symptom recurrence (~20% of original pain).	Noted slight return of burning; still on low-dose amitriptyline.
Month 13	4th ON block (bilateral).	Full remission restored (0% pain); no burning mouth symptoms.
24 Month Follow-up	Continuing follow-up (no further blocks needed in past year).	Sustained complete remission; patient remains essentially asymptomatic.

## Data Availability

The original contributions presented in this study are included in the article. Further inquiries can be directed to the corresponding author.

## References

[1] Jääskeläinen S.K. (2018). Is burning mouth syndrome a neuropathic pain condition?. Pain.

[2] Kouri M., Adamo D., Vardas E., Georgaki M., Canfora F., Mignogna M.D., Nikitakis N. (2024). Small Fiber Neuropathy in Burning Mouth Syndrome: A Systematic Review. Int. J. Mol. Sci..

[3] Zakrzewska J.M., Buchanan J.A. (2016). Burning mouth syndrome. BMJ Clin. Evid..

[4] Scala A., Checchi L., Montevecchi M., Marini I., Giamberardino M.A. (2003). Update on burning mouth syndrome: Overview and patient management. Crit. Rev. Oral Biol. Med..

[5] Walega D.R., Smith C.C., Epstein J.B. (2014). Bilateral stellate ganglion blockade for recalcitrant oral pain from burning mouth syndrome: A case report. J. Oral Facial Pain Headache.

[6] Grémeau-Richard C., Dubray C., Aublet-Cuvelier B., Ughetto S., Woda A. (2010). Effect of lingual nerve block on burning mouth syndrome (stomatodynia): A randomized crossover trial. Pain.

[7] Lee J., Park W., Choi J., Lee G., Ma S., Lee S., Park S. (2023). Effect of repeated greater occipital nerve block in patients with ocular neuropathic pain: A retrospective study. J. Clin. Med..

[8] Treede R.D., Rief W., Barke A., Aziz Q., Bennett M.I., Benoliel R., Cohen M., Evers S., Finnerup N.B., First M.B. (2019). Chronic pain as a symptom or a disease: The IASP Classification of Chronic Pain for the International Classification of Diseases (ICD-11). Pain.

[9] Forssell H., Jääskeläinen S.K., Teerijoki-Oksa T., Hietanen J. (2002). Sensory dysfunction in burning mouth syndrome. Pain.

[10] Kolkka-Palomaa M., Jääskeläinen S.K., Laine M.A., Teerijoki-Oksa T., Sandell M., Forssell H. (2015). Pathophysiology of primary burning mouth syndrome with special focus on taste dysfunction: A review. Oral Dis..

[11] Tan H.L., Smith J., Hoffman J., Renton T. (2021). A systematic review of treatment for patients with burning mouth syndrome. Cephalalgia Rep..

[12] Headache Classification Committee of the International Headache Society (IHS) (2018). The International Classification of Headache Disorders, 3rd edition (ICHD-3). Cephalalgia.

[13] Sun A., Wu K.M., Wang Y.P., Lin H.P., Chen H.M., Chiang C.P. (2013). Burning mouth syndrome: A review and update. J. Oral Pathol. Med..

[14] Piovesan E.J., Kowacs P.A., Oshinsky M.L. (2003). Convergence of cervical and trigeminal sensory afferents. Curr. Pain Headache Rep..

[15] Nasri-Heir C., Zagury J.G., Thomas D., Ananthan S. (2015). Burning mouth syndrome: Current concepts. J. Indian Prosthodont. Soc..

[16] Bartoshuk L.M., Grushka M., Duffy V.B., Fast K., Lucchina L. (1999). Burning mouth syndrome: Damage to CN VII and pain phantoms in CN V. Chem. Senses.

[17] Eliav E., Kamran B., Schaham R., Czerninski R., Gracely R.H., Benoliel R. (2007). Evidence of chorda tympani dysfunction in patients with burning mouth syndrome. J. Am. Dent. Assoc..

[18] Cuadrado M.L., Aledo-Serrano Á., Navarro P., López-Ruiz P., Fernández-De-Las-Peñas C., González-Suárez I., Orviz A., Fernández-Pérez C. (2017). Short-term effects of greater occipital nerve blocks in chronic migraine: A double-blind, randomised, placebo-controlled clinical trial. Cephalalgia.

[19] McMillan R., Forssell H., Buchanan J.A.G., Glenny A.M., Weldon J.C., Zakrzewska J.M. (2016). Interventions for treating burning mouth syndrome. Cochrane Database Syst. Rev..

[20] Lauria G., Majorana A., Borgna M., Lombardi R., Penza P., Padovani A., Sapelli P. (2005). Trigeminal small-fiber sensory neuropathy causes burning mouth syndrome. Pain.

